# Regulation of NDR1 activity by PLK1 ensures proper spindle orientation in mitosis

**DOI:** 10.1038/srep10449

**Published:** 2015-06-09

**Authors:** Maomao Yan, Lingluo Chu, Bo Qin, Zhikai Wang, Xing Liu, Changjiang Jin, Guanglan Zhang, Marta Gomez, Alexander Hergovich, Zhengjun Chen, Ping He, Xinjiao Gao, Xuebiao Yao

**Affiliations:** 1 Anhui Key Laboratory of Cellular Dynamics & Chemical Biology and the University of Science and Technology of China, Hefei 230026, China; 2 Molecular Imaging Center, Morehouse School of Medicine, Atlanta, GA 30310, USA; 3 Shanghai Institute of Biochemistry and Cell Biology, Shanghai 200031, China; 4 UCL Cancer Institute, University College London, London WC1E 6BT, UK; 5 Guangzhou Women and Children’s Medical Center, Guangzhou 510623, China

## Abstract

Accurate chromosome segregation during mitosis requires the physical separation of sister chromatids which depends on correct position of mitotic spindle relative to membrane cortex. Although recent work has identified the role of PLK1 in spindle orientation, the mechanisms underlying PLK1 signaling in spindle positioning and orientation have not been fully illustrated. Here, we identified a conserved signaling axis in which NDR1 kinase activity is regulated by PLK1 in mitosis. PLK1 phosphorylates NDR1 at three putative threonine residues (T7, T183 and T407) at mitotic entry, which elicits PLK1-dependent suppression of NDR1 activity and ensures correct spindle orientation in mitosis. Importantly, persistent expression of non-phosphorylatable NDR1 mutant perturbs spindle orientation. Mechanistically, PLK1-mediated phosphorylation protects the binding of Mob1 to NDR1 and subsequent NDR1 activation. These findings define a conserved signaling axis that integrates dynamic kinetochore-microtubule interaction and spindle orientation control to genomic stability maintenance.

Accurate development of multicellular organism requires well-orchestrated symmetric and asymmetric cell division. Perturbation of symmetry of cell division results in dysmorphia even tumors^1^,^2^. The symmetry of cell division is determined by the division axis relative to the cell polarity axis. In principle, the cell division axis is ruled by mitotic spindle orientation, which is mainly mediated by LGN-NuMA-Dynein-Dynactin signaling axis. In mitosis, extrinsic cues orchestrate LGN-NuMA complex position at cell cortex via dynamic interaction with other cortical polarity proteins. Then LGN-NuMA complex recruits the Dynein-Dynactin complex, a microtubule (MT) minus ends-directed motor complex which can provide pulling forces along astral microtubules to rotate the mitotic spindle^3^. Mounting evidence has demonstrated that several kinase cascades regulated the LGN-NuMA-Dynein-Dynactin signaling axis during mitosis^4^,^5^,^6^. A recent study showed that mitotic kinase PLK1 controled the cortical pulling forces via orchestrating the dynamic interaction between Dynein-Dynactin and LGN-NuMA^7^. However, the precise mechanism underlying PLK1 regulation in spindle orientation has remained to be characterized.

Human NDR1 kinase is a member of evolutionarily conserved NDR family kinases, which play important roles in many biological processes, such as morphological changes, cell proliferation, apoptosis, centrosome duplication and neuron development^8^,^9^,^10^,^11^. Recent studies suggested that NDR1 participated in mitotic process^12^,^13^. However, the precise functions and the underlying regulatory mechanisms remain unclear. Since NDR1 is a primordial kinase, Chiba *et al*. characterized and proposed that Mob2 and Fry synergistically activated NDR1 in mitosis^12^. However, Kohler *et al*. argued that binding of Mob2 in fact interfered with the activation of NDR1^14^. Along this line, Cornils *et al*. has recently shown that the NDR1 activity was much lower in mitosis compared with G1/S phase^15^,^16^.

To explore the molecular mechanism underlying NDR1 function and regulation in mitosis, we characterized the activity profile of NDR1 during cell division. Here we show that persistent activation of NDR1 perturbs spindle orientation. Interestingly, PLK1 interacts with and phosphorylates NDR1 by which NDR1 kinase activity is negatively regulated in mitosis. Thus, our study provides a novel insight into the temporal control of NDR1 kinase activity by PLK1-elicited phosphorylation in mitosis.

## Results

### Persistent activation of NDR1 perturbs mitotic spindle orientation

**Figure 1:**
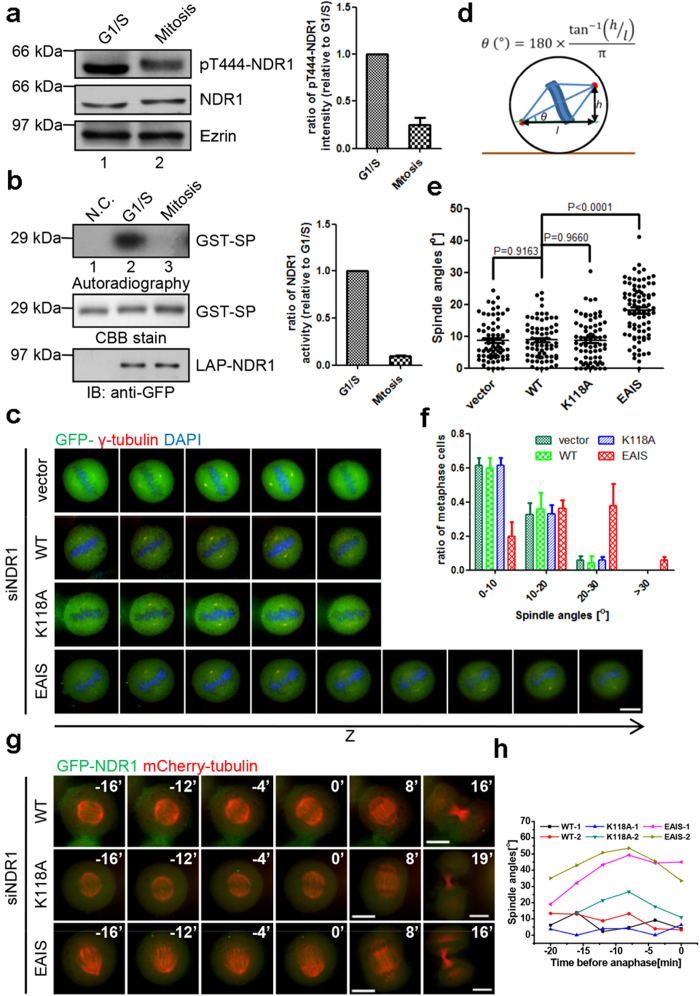
Persistent high-activity of NDR1 disturbs proper spindle orientation. (**a**) Immunoblot analysis of synchronized HeLa cell lysates with antibodies against indicated proteins. Quantification is shown on the right. Bars represent means ± SD from 4 independent experiments. Three cropped panels were taken from same gel and blot. (**b**) Kinase activity measure of LAP-NDR1 purified from synchronized LAP-NDR1 stably expressing HeLa cells or normal HeLa cells (N.C.) *in vitro* kinase assay. The kinase activities were evaluated by autoradiography. The substrate (GST-SP) and kinase (LAP-NDR1) inputs were tested by Coomassie Brilliant Blue (CBB) stain and immunoblot with GFP antibody, respectively. Quantification is shown on the right. Bars represent means ± SD from 3 independent experiments. Cropped gel for CBB stain and autoradiography, cropped LAP-NDR1 blot from another gel. (**c**) Immunofluorescence analyses of mitotic HeLa cells transfected with siRNA-resistant indicated constructs together with NDR1 siRNA. Cells were synchronized at G1/S phase by thymidine, and then fixed at 9.5 hours after release, stained with γ-tubulin antibody (red) and DAPI (blue). Z-sections images (0.4 μm per stack) are shown at the interval of two stacks. Scale bar represents 10 μm. (**d**) Schematic diagram and the formula for calculation of spindle angles. *θ* represents the spindle angle, *h* and *l* represent the vertical and horizontal difference of the two spindle poles (indicated by γ-tubulin), respectively.(**e**) Scatter plots of the spindle angles of metaphase cells shown in (**c**). Bars indicate means±SEM from analyses of more than 80 cells from three independent experiments. Two-tailed student’s *t*-test for *p*-value calculation.(**f**) Ratio of metaphase cells within the indicated angle sections as described in (**e**). Bars indicate means±SD from three independent experiments.(**g**) Time-lapse imaging of HeLa cells transfected with NDR1 siRNA, mCherry-tubulin plus GFP-tagged siRNA-resistant NDR1 constructs. Time-lapse images at a fixed z-stack were shown at the indicated time points (min) relative to anaphase onset. Images were acquired within 0.8 μm per stack for each time point. Scale bars represent 10 μm.(**h**) Time-lapse spindle angles of mitotic cells in (**g**). Two cells for each construct. The positions of spindle poles were assessed by Cherry fluorescence.

Early studies suggest the role of NDR1 in chromosome movement in mitosis^12^. However, the precise mechanism underlying NDR1 regulation is unclear. To delineate the precise function of NDR1 in mitosis, we attempted to characterize the NDR1 activity in mitotic cells. Consistent with previous studies^15^,^16^, we found that the phosphorylation of NDR1 on Thr444, a readout of NDR1 kinase activity, is much lower in mitosis than that in interphase judged by quantitative Western blotting (Fig. 1a; p < 0.001). To confirm the NDR1 activity suppression in mitosis, we also isolated NDR1 kinase from mitotic and interphase cells and compared their kinase activities *in vitro*. To this end, aliquots of HeLa cells stably expressing LAP-tagged NDR1 (referred as GFP-S-NDR1) were synchronized and collected at interphase (G1/S) and mitosis. Equal amounts of purified NDR1 from HeLa cells were then incubated with NDR1 substrate (GST-SP) in the presence of ^32^P-γ-ATP. As shown in Fig. 1b, NDR1 isolated from interphase cells exhibited about 10-fold higher activity than that of mitotic NDR1 (lanes 2 and 3). Thus, we conclude that NDR1 kinase activity is suppressed in mitosis.

If NDR1 kinase activity were negatively regulated in mitosis, persistent expression of active NDR1 would perturb accurate mitosis. Previous study has shown that an NDR1 mutant (NDR1^EAIS^), in which five positively charged amino acids of its auto-inhibitory sequence were all mutated to alanine, possessed greatly elevated basal kinase activity like being Mob1-stimulated^17^. Consistently, in our study, we demonstrated that NDR1^EAIS^ kept high-level activity during mitosis (Supplementary Fig. 1b). Accordingly, this mutant can be used as a constitutively active mutant. Subsequent immunofluorescence microscopic analyses were performed to check whether persistent expression of GFP-NDR1^EAIS^ modulates mitotic progression. Aliquots of HeLa cells were transiently transfected to express siRNA-resistant GFP-tagged NDR1 wild-type (NDR1^WT^), dominant negative (NDR1^K118A^) or constitutive active (NDR1^EAIS^) mutant together with NDR1 siRNA to suppress endogenous NDR1 levels. Western blotting analysis of transfected cells showed that exogenously expressed NDR1 proteins were about 3-fold of the level of endogenous NDR1 in HeLa cells (Supplementary Fig. 1a). Transfected cells were co-stained for spindle using an anti-tubulin antibody (*red*) and for centromere labeling using anti-centromere auto-antibody (ACA; *white*). The DNA was labeled with DAPI (*blue*). Surprisingly, as shown in Supplementary Fig. 1c, chromosomes from all three different groups appeared well aligned. However, careful examination of mitotic spindle geometry showed that in NDR1^EAIS^-expressing cells, the projection width (PW) of chromosome alignment was significantly larger than the single layer width (SW) of chromosome alignment, but in NDR1^WT^ or NDR1^K118A^-expressing cells, whose NDR1 activities were expectantly low, the PW was very close to the SW (Supplementary Fig. 1c and 1d). This suggested a greater degree of orientation in NDR1^EAIS^-expressing cells. Interestingly, although depletion of NDR1 led to chromosome misalignment in many cells^12^, the value of PW was almost equal to that of SW in the relatively normal vector-transfected cells (Supplementary Fig. 1c and 1d). Quantification of the spindle angles in transfected cells supports the notion that persistent expression of NDR1^EAIS^ promotes spindle rotation (Supplementary Fig. 1e).

To further verify the relationship between NDR1 activity and spindle orientation, we next examined the positions of spindle poles in transfected cells marked by γ-tubulin antibody. We found that both spindle poles marked by γ-tubulin were often localized in the same focal plane through 4-6 adjacent optical sections in the transfected cells bearing low NDR1 activity. However, neither of spindle poles was observed in a same focal plane in NDR1^EAIS^-expressing cells (Fig. 1c). In addition, the two spindle poles marked by γ-tubulin were distributed in a much broader range of z-sections in NDR1^EAIS^-expressing cells. These results suggest that the mitotic spindle is aberrantly oriented in those cells with persistent high-activity of NDR1.

To precisely characterize the spindle tilt, mitotic spindle angles were quantified using the formula illustrated in Fig. 1d. As expected, the spindle tilt in NDR1^EAIS^-expressing cells was much greater than those in other groups (Fig. 1e). Quantitative analyses showed that, consistent with previous studies^18^,^19^, the average spindle angles in transfected cells bearing low NDR1 activity (vector, WT and K118A) were all less than 10 degrees, while the degree of spindle rotating angle in NDR1^EAIS^-expressing cells was over 20 degrees (p < 0.01; n = 100) (Fig. 1e). Interestingly, suppression of NDR1 led to chromosome misalignment without alteration of spindle orientation while persistent expression of active NDR1 led to aberrant spindle orientation. In fact, quantitative analyses of spindle angles distribution showed that the percentage of the cells with large angle (>10^o^) increased to a great extent when expressing constitutive active NDR1^EAIS^ (Fig. 1f). Thus, we conclude that precise regulation of NDR1 kinase activity is essential for accurate mitosis.

As the mitotic spindle undergoes a certain extent of rotation to facilitate the kinetochore capture before metaphase, it is therefore necessary to rule out the possibility that this spindle tilt is a consequence of potential mitotic delay. To this end, we monitored the mitotic spindle in the real-time imaging. Consistent with previous results from immunofluorescence analyses, NDR1^EAIS^-expressing cells manifested larger spindle angles at the anaphase onset than control cells (vector, WT and K118A; Fig. 1h). Together, we conclude that low activity of NDR1 in mitosis is necessary for proper spindle orientation.

### PLK1 interacts with and phosphorylates NDR1

**Figure 2:**
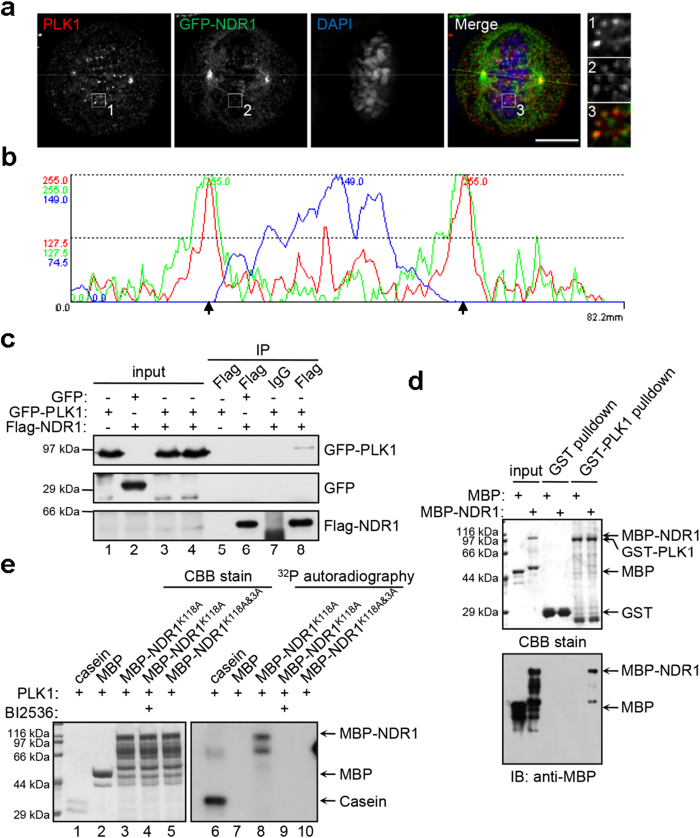
PLK1 interacts with and directly phosphorylates NDR1 (a) Co-localization of NDR1 and PLK1 in mitosis. Immunofluorescence analyses of mitotic HeLa cells expressing GFP-NDR1 wild-type construct with PLK1 antibody (red) and DAPI (blue). The boxed areas are shown in the right row with magnification. The scale bar represents 10 μm.(**b**) Line scan of the yellow straight line in the “merge” pane of (**a**). Black arrowheads indicate the spindle poles.(**c**) Co-immunoprecipitation of Flag-NDR1 and GFP-PLK1. Lysates from the 293T cells transiently transfected to express GFP-PLK1 alone, or FLAG-NDR1 together with GFP or GFP-PLK1 were subjected to immunoprecipitation with Flag antibody or IgG. The precipitates (IP) and cell lysates were analyzed by immunoblot with antibodies against Flag and GFP. Cropped blots from one gel.(**d**)Pull-down assay of MBP-NDR1 using GST- or GST-PLK1-bound agarose beads as an affinity matrix.(**e**) *In vitro* kinase assay with using PLK1 kinase mixed with substrates of MBP, MBP-NDR1 kinase-death mutant (K118A) and non-phosphorylatable mutant (K118A-3A). In some case, an aliquot of reaction included PLK1 specific inhibitor BI2536. Both CBB stain gel and autoradiography are shown.

During mitosis, both Mst1/2 and Mob1, the most powerful and intimate NDR1 activators^9^,^11^,^17^,^20^,^21^,^22^,^23^, have been reported to exhibit an increase in activity and/or abundance during mitosis. Hence, there should be an inhibitory mechanism underlying the suppression of NDR1 in mitosis. We noticed that NDR1 protein from mitotic cells exhibited a typical mobility shift in SDS-PAGE and related Western blotting (Fig. 1a; lane 2), suggesting that NDR1 was modified other than phosphorylation on Thr444. Recently, emerging evidence indicates PLK1 is involved in the regulation of spindle orientation in addition to its classical role in chromosome segregation^7^,^18^. Recent structure-based prediction of protein-protein interaction suggests that NDR1 binds to PLK1^24^. Thus, we sought to examine whether PLK1 interacts with and regulates NDR1. As shown in Fig. 2a, PLK1 was apparently co-localized with NDR1 at centrosome/spindle poles, kinetochores, and even in cytoplasm. Line scan analyses demonstrate the co-localization profile of PLK1 and NDR1 (Fig. 2b), supporting the notion that PLK1 interacts with NDR1. To confirm their interaction, we carried out an immunoprecipitation experiment using 293 T cells transiently transfected to express GFP-PLK1 and FLAG-NDR1. As shown in Fig. 2c, GFP-PLK1, but not GFP, was pulled down by FLAG-NDR1 judged by western blotting (lane 8), confirming that exogenously expressed GFP-PLK1 forms a complex with FLAG-NDR1.

To validate whether PLK1 directly interacts with NDR1, we employed GST-PLK1 fusion protein as affinity matrix and applied MBP-tagged NDR1 as an input. As shown in Fig. 2d, MBP-NDR1, but not MBP, was retained by GST-PLK1 rather than GST-bound affinity matrix, demonstrating a direct and physical interaction between PLK1 and NDR1.

After demonstrating a physical link between PLK1 and NDR1, we sought to examine whether PLK1 phosphorylates NDR1. To this end, aliquots of MBP fusion proteins containing NDR1 mutants were incubated with PLK1 in the presence of ^32^P-γ-ATP with or without PLK1 inhibitor BI2536^25^. As shown in Fig. 2e, PLK1 effectively phosphorylated MBP-NDR1^K118A^ but not MBP judged by the incorporation of ^32^P (lane 8). The incorporation of ^32^P into NDR1 was diminished by PLK1 inhibitor BI2536. To identify the potential PLK1-elicited phosphorylation sites on NDR1, we used GPS 2.1, a powerful tool for phosphorylation sites prediction reported previously^26^, and identified three potential sites (Thr7, Thr183 and Thr407) as potential PLK1 substrates (Supplementary Fig. 2). As shown in Fig. 2e (lane 10), mutation of Thr7, Thr183 and Thr407 into none-phosphorylatable alanines (MBP-NDR1^K118A&3A^) abolished the incorporation of ^32^P into NDR1 protein. Taken together, we conclude that PLK1 interacts with and phosphorylates NDR1.

### Suppression of NDR1 by PLK1-elicited phosphorylation ensures proper spindle orientation

**Figure 3:**
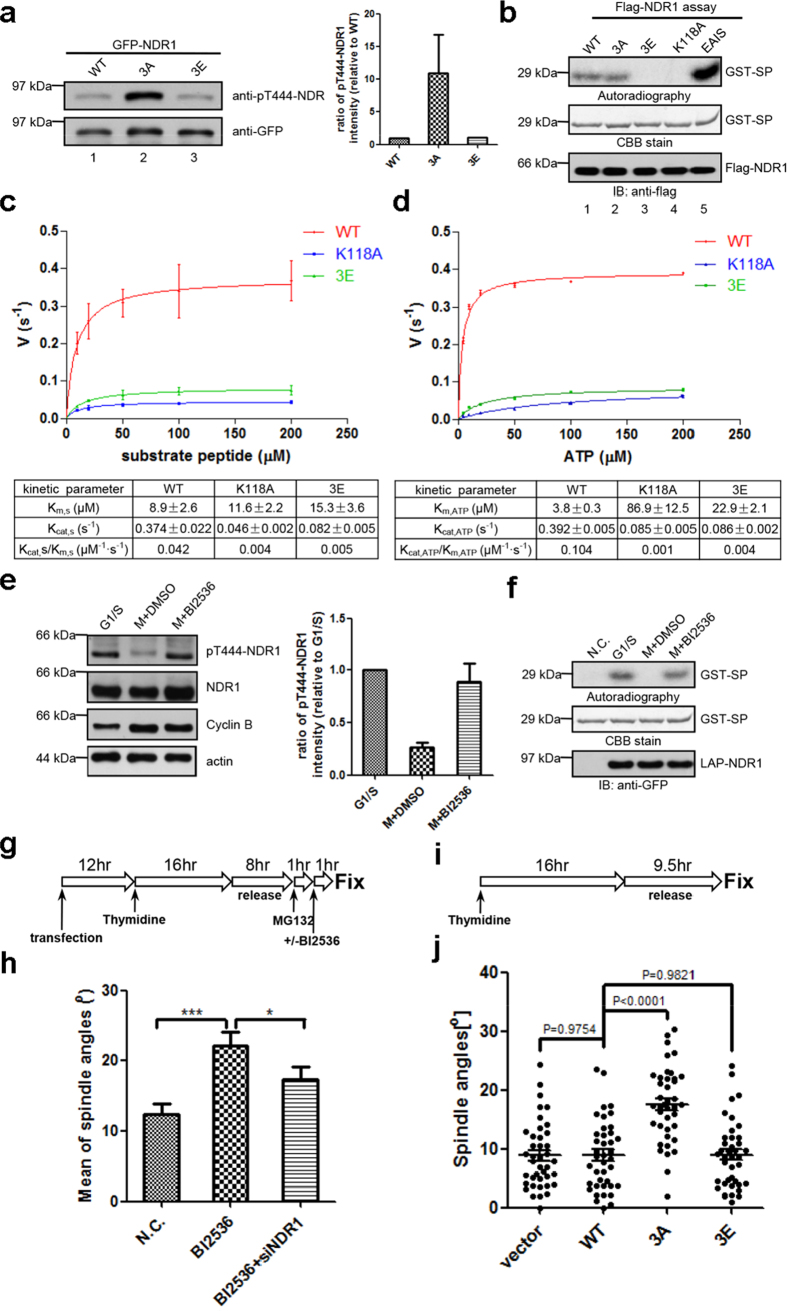
PLK1-mediated phosphorylation suppressed NDR1 activity to ensure proper spindle orientation (a) Immunoblot of mitotic HeLa cell lysates expressing GFP-NDR1^WT^, GFP-NDR1^3A^ or GFP-NDR1^3E^, with anti-GFP and anti-pT444-NDR1 antibodies. Quantification is shown on the right. Bars represent means ± SD, n = 3. (**b**) Kinase activity measure of Flag-NDR1 wild-type or mutants (3A, 3E, K118A and EAIS) purified from asynchronized 293 T cells *in vitro* kinase assay. Full-length blots/gels are presented in Supplementary Fig. S4.(**c**)and (**d**) Determination of kinetic parameters of NDR1^WT^, NDR1^K118A^ and NDR1^3E^. The velocities of kinase assay toward the 10-mer substrate peptide (**c**) or ATP (**d**) at varying concentrations were measured by Amplite^TM^ Universal Fluorimetric Kinase Assay Kit. Data from three independent experiments were analyzed in GraphPad Prism and fitted with Michaelis-Menten equation to extract the kinetic parameters. Bars indicate means±SD.(**e**) Immunoblot analyses of synchronized HeLa cell lysates with (+BI2536) or without (+DMSO) BI2536 treatment, with the antibodies against indicated proteins. “M” represents mitosis. Quantification is shown on the right. Bars represent means±SD from 3 independent experiments. Full-length blots/gels are presented in Supplementary Figure S4.(**f**) Kinase activity measure of LAP-NDR1 purified from synchronized LAP-NDR1 stable expressing HeLa cells with (+BI2536) or without (+DMSO) BI2536 treatment, or normal HeLa cells (N.C.) *in vitro* kinase assay. Full-length blots/gels are presented in Supplementary Figure S4.(**g**) Schematic overview of the experimental procedure of (**h**). (**h**) Quantification of spindle angles of mitotic HeLa cells expressing NDR1 siRNA (+siNDR1) or not, with or without BI2536 treatment, as described in (**g**). Immunofluorescence images were obtained and analyzed as in Fig. 1e. “N.C.” represents cells with neither transfection nor BI2536 treatment. Bars indicate means±SEM from analyses of no less than 40 cells with deformed spindles. Two-tailed student’s *t*-tes*t* for *p*-value calculation.(**i**) Schematic overview of the experimental procedure of (**j**).(**j**) Scatter plots of spindle angles in mitotic HeLa cells transfected with NDR1 siRNA plus various siRNA-resistant NDR1 constructs, as described in (**i**). Immunofluorescence images were obtained and analyzed as in Figure 1e. Bars indicate means±SEM from analyses of 40 cells. Two-tailed student’s *t*-test for *p*-value calculation.

We next examined whether PLK1-elicited phosphorylation at Thr7, Thr183 and Thr407 impairs NDR1 kinase activity. To this end, aliquots of HeLa cells were transiently transfected to express GFP-NDR1^WT^, GFP-NDR1^3A^ and GFP-NDR1^3E^ followed by nocodazole treatment to synchronize the aforementioned cells in mitosis. Soluble cell lysates from HeLa cells expressing GFP-NDR1^WT^, GFP-NDR1^3A^ and GFP-NDR1^3E^ were generated and subjected to Western blotting analyses of NDR1 activity using pT444 antibody as readout. As shown in Fig. 3a, GFP-NDR1^WT^, GFP-NDR1^3A^ and GFP-NDR1^3E^ expressed at comparable levels in HeLa cells judged by GFP Western blotting (bottom panel). However, examination of pT444 blotting revealed that the level of NDR1 phosphorylation at T444 in HeLa cells expressing GFP-NDR1^3A^ was about 11 fold higher than that in GFP-NDR1^WT^-expressing cells (lanes 1 and 2). If PLK1-elicited phosphorylation suppressed NDR1 activity, phospho-mimicking mutant NDR1^3E^ would exhibit a similar low activity profile like that of wild type NDR1. Consistent with our speculation, the level of T444 phosphorylation in HeLa cells expressing GFP-NDR1^3E^ was equivalent to that in NDR1^WT^-expressing cells (Fig. 3a, lane 3). Quantitative analyses showed that the level of pT444 in GFP-NDR1^3A^ was significantly higher than those of wild type and GFP-NDR1^3A^-expressing cells (Fig. 3a, right graph; p < 0.001), suggesting that PLK1-elicited phosphorylation may regulate NDR1 kinase activity.

To directly examine the NDR1 kinase activity in response to PLK1-mediated phosphorylation, we carried out an *in vitro* phosphorylation experiment in which aliquots of NDR1 substrate peptide were incubated with equal amount of various FLAG-NDR1 kinase proteins isolated from 293 T cells. The substrate peptide does not contain consensus site for PLK1 kinase so the phosphorylation is an accurate and specific reporter for NDR1 kinase activity *in vitro*. As shown in Fig. 3b, Flag-NDR1^EAIS^ exhibited a prominent kinase activity judged by ^32^P incorporation into peptide substrate (top panel, lane 5). It was readily apparent that PLK1 phospho-mimicking mutant GFP-NDR1^3E^ exhibited undetectable kinase activity (lane 3). On the other hand, FLAG-NDR1^3A^ exhibited apparent kinase activity judged by the ^32^P incorporation into the substrate (lane 2). Since NDR1 was purified from unsynchronized 293 T cells, which contains low PLK1 kinase activity, FLAG-NDR1^WT^ also displayed kinase activity similar to FLAG-NDR1^3A^ (lane 1). Thus, we conclude that PLK1-elicited phosphorylation suppresses NDR1 kinase activity.

To delineate the mechanism underlying PLK1-elicited NDR1 kinase inhibition, we then carried out enzymatic assay and measured the kinetic parameters of recombinant NDR1 wild-type, kinase-dead and phospho-mimetic mutant toward two substrates: SP and ATP. As shown in Fig. 3c,d (see also Supplementary Fig. 3a), for both substrates, the K_m_ parameter of NDR1^3A^ was larger than that of wild type NDR1 (~1.7-fold toward SP and ~6-fold toward ATP). The maximal velocity of phosphorylation by NDR1^3E^ was about 4.5-fold slower, for both substrates, compared with that of NDR1^WT^. These kinetic analyses indicate that phosphorylation of NDR1 by PLK1 not only reduced the catalytic rate but also decreased the affinity between NDR1 and its substrates. To measure the catalytic efficiency, we calculated the ratio of k_cat_/K_m_ of various NDR1 proteins, and found that the phospho-mimicking mutant NDR1^3E^ exhibits much reduced efficiency in phospho-transfer compared to those of NDR1^WT^ (k_cat_/K_m_ is 7.7-fold decreased for SP and ~27.5-fold decreased for ATP). However, the NDR1^3E^ remains its catalytic activity over the kinase-death mutant NDR1^K118A^. Thus, we conclude that PLK1-elicited phosphorylation reduces the ability of NDR1 to catalyze phosphate transfer to its substrates.

We next examined whether PLK1-elicited phosphorylation is responsible for NDR1 activity reduction in mitosis. Careful examination of GFP-NDR1^WT^ and GFP-NDR1^3E^ isolated from mitotic HeLa cells indicated that wild type NDR1 exhibits comparable activity equivalent to PLK1-phosphorylated NDR1 (Fig. 3a; lanes 1 and 3), suggesting that wild-type NDR1 is likely phosphorylated during mitosis. If this were correct, inhibition of PLK1 by chemical inhibitor would resume NDR1 activity. Consistent with our hypothesis, treatment of mitotic cells with PLK1 inhibitor BI2536 fully released the inhibition of endogenous NDR1 kinase activity in mitosis (Fig. 3e,f). Thus, we conclude that NDR1 activity in mitosis is governed by PLK1-mediated phosphorylation.

Next, we examined the precise role of PLK1-mediated phosphorylation of NDR1 in mitotic spindle position control. To this end, we took the advantage of creating unstable astral microtubule and rotating spindle using an accurate treatment of PLK1 chemical inhibitor BI2536 (illustrated in supplementary Fig. 4). Quantitative analyses indicate that the chemical inhibition of PLK1 dramatically increased the mitotic spindle angles (Fig. 3g,h), validating that PLK1 kinase activity is essential for orchestrating accurate spindle orientation^7^,^19^,^27^. Since PLK1 controls spindle orientation via NDR1-dependent and NDR1-independent pathways, we sought to test whether the perturbation of spindle positioning in BI2536-treated cells is somewhat compromised in the absence of NDR1. As predicted, depletion of NDR1 has attenuated the BI2536-elicited spindle orientation defects (Fig. 3g,h), indicating that NDR1 is an important downstream effector underlying PLK1 signaling in spindle position control.

To further demonstrate the relevance of PLK1-NDR1 signaling in spindle orientation, aliquots of GFP-NDR1^3A^ and control plasmids were transiently transfected into HeLa cells followed by quantifying spindle angles of mitotic cells. As shown in Fig. 3i,j, persistent expression of unrestrained GFP-NDR1^3A^ significantly increased the spindle orientation angles compared to those of GFP-NDR1^WT^ and GFP-NDR1^3E^ (about 2-fold increase; p < 0.001). Importantly, this change in spindle orientation was not a consequence of alteration of PLK1 kinase activity feedback by NDR1 activation because neither expression nor depletion of NDR1 kinases altered PLK1 kinase activity judged by the level of phosphorylation of PLK1 on Thr210 (Supplementary Fig. 3b)^28^,^29^. Thus, we conclude that PLK1-mediated phosphorylation of NDR1 is essential for proper spindle orientation control.

### PLK1-mediated phosphorylation interferes with the activation of NDR1 by Mob1

**Figure 4:**
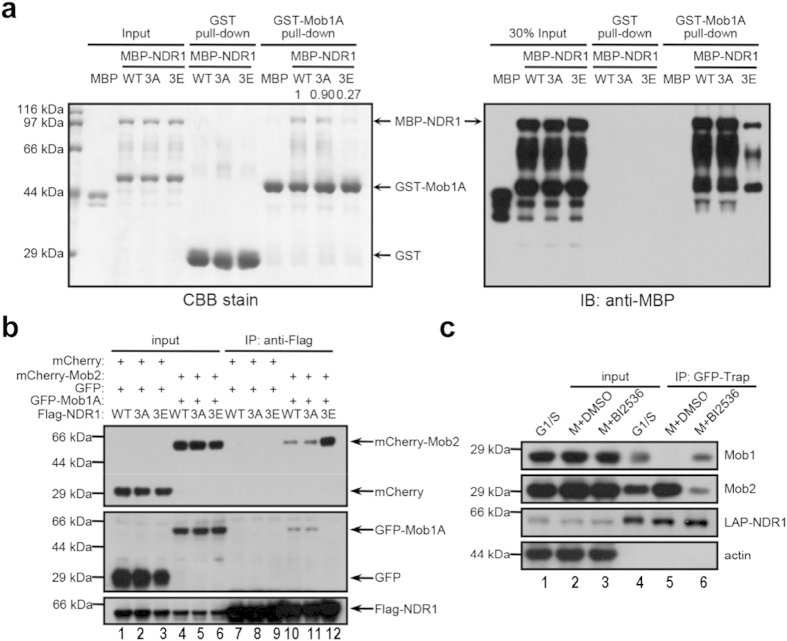
PLK1 restricts NDR1 kinase activity through interfering with activator Mob1-binding (a) Pull-down assay of MBP-NDR1 wild-type or mutants (WT, 3A and 3E) using GST- or GST-Mob1A-bound agarose beads. The pulled-down MBP-NDR1 bands were quantified by Image J software and normalized. (**b**) Co-immunoprecipitation of Flag-NDR1 (WT, 3A and 3E) with GFP-Mob1A and mCherry-Mob2. Lysates of asynchronized 293 T cells expressing indicated plasmids were subjected to immunoprecipitation with Flag antibody. The precipitates (IP) and cell lysates were analyzed by immunoblot with antibodies against Flag, GFP and Cherry, respectively. (**c**) Co-immunoprecipitation of LAP-NDR1 with endogenous Mob1 and Mob2. LAP-NDR1 stable expressing HeLa cells were treated as in Fig. 3h. The lysates were subjected to immunoprecipitation with GFP-Trap agarose beads. The precipitates (IP) and cell lysates were analyzed by immunoblot with antibodies against GFP, actin, Mob1 and Mob2. Cropped blots from same gel.

After the demonstration of PLK1-elicited suppression of NDR1 activity, we sought to explore the mechanism underlying this inhibition. Previous studies have demonstrated the crucial role of Mob1-binding in NDR1 activation^17^,^20^,^30^, we wondered whether PLK1-mediated phosphorylation alters the Mob1-binding. To this end, we carried out a pull-down assay to examine how phosphomimicking NDR1 mutants bind to recombinant Mob1A relative to wild type protein. As shown in Fig. 4a, phosphomimetic mutant NDR1^3E^ apparently reduced the binding to Mob1A while non-phosphorylatable NDR1^3A^ bound to Mob1A in a similar extent to that of wild type NDR1. Densitometric analyses of MBP fusion proteins retained on GST-Mob1 affinity matrix indicated that, relative to NDR1^WT^, less than 30% of phosphomimetic mutant NDR1^3E^ bound to Mob1 while great than 90% NDR1^3A^ was retained by the Mob1 (Fig. 4a). To test whether PLK1-mediated phosphorylation alters NDR1 interaction with Mob1 in cell lysates, we carried out immunoprecipitation in which 293 T cells were transiently transfected to express mCherry-Mob2, GFP-Mob1A and various FLAG-NDR1 mutants. It has been proposed that Mob2 and Mob1 competitively binds to NDR kinases and Mob2-binding interferes with Mob1-elicited activation of NDR kinase^14^. Consistent with previous reports, much more Mob2 proteins were detected in those precipitates containing lesser Mob1 proteins (Fig. 4b), suggesting that PLK1-elicited phosphorylation induces a conformational change of NDR1, which promotes Mob2 binding to NDR1. Consistent with this rationale, addition of PLK1 chemical inhibitor into mitotic HeLa cells restored Mob1 binding to NDR1 (Fig. 4c). Thus, we conclude that PLK1-mediated phosphorylation of NDR1 switches its binding from Mob1 to Mob2 which abolishes Mob1-elicited NDR1 activation.

### PLK1 phosphorylation on NDR1 is necessary for the cortical localization of LGN-NuMA complex

**Figure 5:**
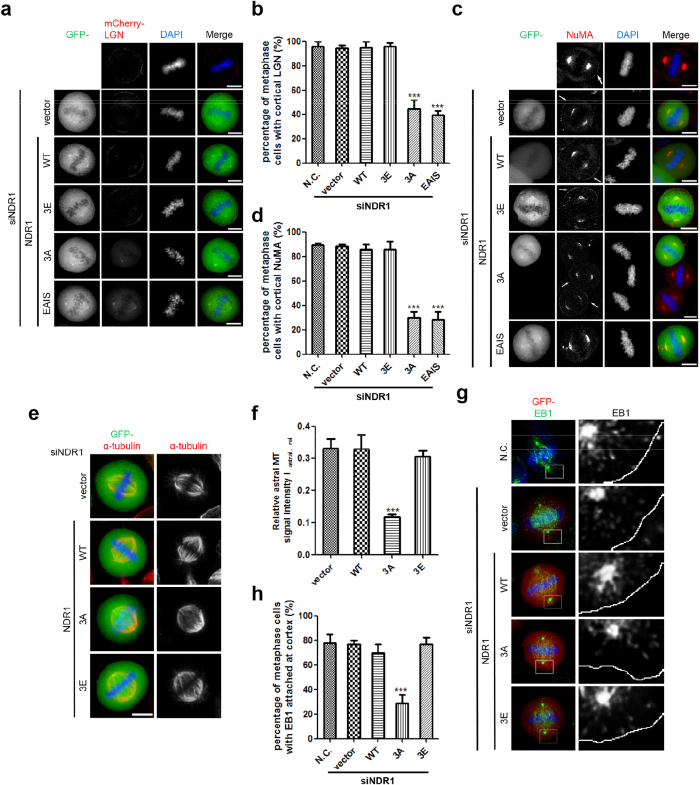
Phosphorylation of NDR1 by PLK1 is essential for cortical localization of LGN-NuMA (a) Immunofluorescence analyses of mitotic HeLa cells expressing mCherry-LGN and GFP-NDR1 wild-type or mutants (WT, 3A or 3E) together with NDR1 siRNA. The scale bar represents 10 μm.(**b**) Percentage of metaphase cells with obvious enrichment of LGN at cell cortex as in (**a**). Bars indicate means±SD, n = 3, 40 cells for each experiment. The significant difference is evaluated by two-tailed student’s *t*-test and labelled.(**c**)Immunofluorescence analyses of mitotic HeLa cells expressing GFP-NDR1 wild-type or mutants (WT, 3A or 3E) together with NDR1 siRNA, using NuMA (red) antibody. In the 3A panel, the GFP positive cell indicates the transfected cell. The scale bar represents 10 μm. (**d**) Percentage of metaphase cells containing cortical NuMA signal as in (**c**). Bars indi**c**ate means ± SD, n = 3, 40 cells for each experiment. The significant difference is evaluated by two-tailed student’s *t*-test and labelled.(**e**) Immunofluorescence analyses of mitotic HeLa cells expressing GFP-NDR1 wild-type or mutants (WT, 3A or 3E) together with NDR1 siRNA, using α-tubulin antibody. The scale bar represents 10 μm.(**f**) Quantification of the relative signal intensities of astral microtubules compared to spindle microtubules as in (**c**). Bars indi**c**ate means±SEM from analyses of 30 cells. The significant difference is evaluated by two-tailed student’s *t*-test and labelled.(**g**) Immunofluorescence analyses of mitotic HeLa cells expressing GFP-NDR1 wild-type or mutants (WT, 3A or 3E) together with NDR1 siRNA, using EB1 antibody. One z-stack was shown for each construct. In the lower panel, EB1 signal channel was shown as black-and-white image, and the cell border was shown as white solid-line curve. The scale bar represents 10 μm.(**h**) Percentage of metaphase cells with EB1 signal reached cell cortex as in (**g**). Bars indicate means±SD, n = 3, 40 cells for each experiment. The significant difference is evaluated by two-tailed student’s *t*-test and labelled.

Accurate spindle orientation determines cell polarity and cell fate in tissue plasticity and homeostasis^1^,^31^,^32^. In HeLa cells, to achieve proper spindle orientation, principally, except for spindle poles localization^33^,^34^, LGN-NuMA symmetrically distributes at the cell cortex for recruitment of dynein-dynactin complex^3^,^35^. Dynein-dynactin complex coordinates the astral microtubule to plasma membrane cortex by which it generates pulling forces to rotate the spindle for optimal capture of kinetochore. To determine how persistently active NDR1 disturbs spindle orientation, we first examined the localization of LGN/NuMA in cells expressing wild type or mutated NDR1 proteins. As shown in Fig. 5a-d, only a little part of cells expressing GFP-NDR1^3A^ or GFP-NDR1^EAIS^ displayed an obvious enrichment of either LGN (~44% for GFP-NDR1^3A^ and ~39% for GFP-NDR1^EAIS^) or NuMA (~30% for GFP-NDR1^3A^ and ~28% for GFP-NDR1^EAIS^) at the cell cortex. In particular, as shown in Fig. 5c, the cortical localization of NuMA was readily apparent in the cells failed to be transfected to express GFP-NDR1^3A^, while in nearby cells positively transfected to express GFP-NDR1^3A^ the cortical signal of NuMA localization was diminished. Thus, we conclude that persistent expression of active NDR1 perturbs the cortical targeting and/or the maintenance of LGN-NuMA localization.

Anchorage of astral microtubule plus-ends to the cortex is required for its stabilization and proper spindle orientation, which is mediated by the interactions between astral microtubules and cortical proteins including LGN-NuMA complex^3^. Thus, persistent activation of NDR1 activity, which leads to delocalization of LGN-NuMA, should disturb the linkage between astral microtubules and cell cortex, thereby destabilize and shorten the astral microtubules. Consistently, we found that expression of GFP-NDR1^3A^ reduced the relative astral microtubules intensity to about 40% of that in GFP-NDR1^WT^-expressing cells (Fig. 5e,f), suggesting that persistent activation of NDR1 perhaps promotes microtubule destabilization. To monitor the cortical anchorages of astral microtubules, we stained the mitotic cells for EB1, a microtubule+TIP protein marking the astral microtubule ends. We found that EB1 reached the cell cortex in majority of cells with low NDR1 activities (N.C., vector, WT and 3E), whereas only ~29% of GFP-NDR1^3A^-expressing mitotic cells showed EB1 signal near the cell cortex (Fig. 5g,h). Thus, we conclude that the persistent activation of NDR1 disturbs proper spindle orientation through delocalizing LGN-NuMA complex from the cell cortex.

## Discussion

Faithful genome segregation depends on the plasticity and dynamic regulation of mitotic spindle. Molecular delineation of the mechanisms that control spindle position and orientation is key to understand cell division control. Here, we demonstrated that NDR1 kinase activity is dynamically regulated in cell division by PLK1 to correct spindle position and orientation in symmetrically dividing human cells. Plk1 negatively regulates NDR1 kinase activity by switching from its activator Mob1-binding to Mob2-binding, which restricts NDR1 kinase activation to ensure accurate spindle orientation. Our findings help to resolve the controversy surrounding NDR1 activity regulation in mitosis^12^,^13^,^14^,^15^,^16^. Future work will be required to define the molecular basis underlying the aforementioned PLK1-elicited NDR1-Mob2 interaction and delineate the respective role of each of three phosphorylation sites in mediating NDR1-binder switch.

Previous study has reported that suppression of NDR1 resulted in chromosome misalignment but failed to uncover the underlying mechanisms^12^. In addition, a recent study argued that HP1α phosphorylated by NDR1 kinase is required for mitotic progression and for Sgo1 binding to mitotic centromeres^36^. Together with our recent study showed that mitotic kinases such as PLK1 and TTK regulate the cross-talk between microtubules and F-actin filaments in ensuring a correct spindle position and symmetric division^27^, it is of great importance to illustrate the spatiotemporal dynamics of NDR1 kinase activity gradient to further elucidate its dynamic gradient reated to that of PLK1. The cross-talk of PLK1 and NDR1 signaling at the spindle pole and its relation to the spindle geometry can be monitored and quantified using FRET-based activity reporter localized to the spindle pole and cortex^37^. This line of experimentation will also validate the outcome generated from the complex experimentation in which NDR1 expression level was suppressed while PLK1 activity is inhibited by chemical inhibitor.

**Figure 6:**
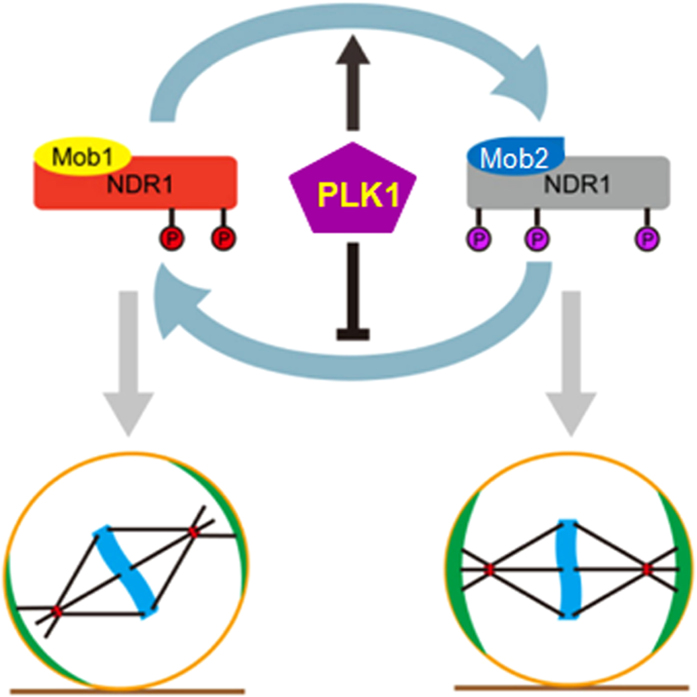
Working model accounting for PLK1-NDR1 signaling in mitotic spindle orientation control Kinase regulators Mob1 and Mob2 competitively bind NDR1. Mob1-bound NDR1 is active with phosphorylation on Ser281 and Thr444, while Mob2-bound NDR1 is inactive. In normal mitosis, PLK1 phosphorylates NDR1 on three sites (Thr7, Thr183 and Thr407), switching NDR1 from Mob1-binding to Mob2-binding, hence prevents NDR1 from Mob1-elicited activation to ensure proper spindle orientation. In the case of reversal PLK1-elicited suppression of NDR1, the cortical localization of LGN-NuMA is liberated which leads to aberrant mitotic spindle orientation.

Our study revealed that PLK1 negatively regulated NDR1 activity through phosphorylation on three potential sites of threonine residues, which switched NDR1 binding from Mob1 to Mob2. It would be of great interest to delineate how dynamic phosphorylation and dephosphorylation of NDR1 are orchestrated as PLK1-elicited inhibition of NDR1 liberates the LGN-NuMA complex from cell cortex and alters accurate spindle orientation (Fig. 6). PLK1 is enriched at the spindle poles, and it was proposed that there is a gradient of its kinase activity which regulates the NuMA-LGN/Gαi ternary complex plasticity. However, the molecular nature of PLK1 substrates involved in spindle positioning remains to be identified. Future studies will also address whether and how NDR1 regulates NuMA-LGN/Gαi interaction at the cell cortex. Proteomic and chemical genetic identification of NDR1 kinase will establish the functional relationships of these modifications in mitotic orchestration of NDR1 activity in spindle plasticity and orientation control. Better understanding of spindle position control are expected from delineating the spatiotemporal dynamics of the force generation machinery in response to extracellular cues as changes in microenvironment could alter the spindle positioning in development and disease progression^38^.

Taken together, we demonstrate that the evolutionarily conserved NDR1 kinase physically interacts with PLK1 and relays PLK1 signaling in regulation of spindle positioning. The PLK1-elicited phosphorylation of NDR1 negatively regulates NDR1 activity by preventing its binding to activator Mob1. Our findings illustrate a unique molecular mechanism underlying PLK1-dependent inactivation of NDR1 and define a novel role for PLK1-NDR1 signaling cascade in governing accurate spindle orientation to ensure faithful chromosome segregation in mitosis.

## Methods

### Plasmids construction and siRNAs

HeLa total RNA was extracted by the traditional Trizol method. HeLa cDNA library was constructed by RT-PCR using oligo (dT)^18^ and M-MLV Reverse Transcriptase (Invitrogen) from the total RNA. NDR1, Mob1A and Mob2 cDNA were isolated by PCR from HeLa cDNA library. To generate GFP-, Flag- and MBP-tagged full-length human NDR1, PCR-amplified NDR1 cDNA was cloned to pEGFP-C1 (BD Biosciences), p3X Flag-myc-CMV24 (Sigma) and pMAL-C2x (Addgene) vectors using *Bgl*II/*Bam*HI and *Sal*I restriction sites. GST-tagged Mob1A was constructed by cloning PCR-amplified Mob1A cDNA into pGEX-6P-1 (GE Healthcare) vector with *Bam*HI and *Sal*I digestion. To obtain GST-SP that express a NDR1 substrate peptide^39^ fused to GST, the following oligonucleotide pair was inserted into pGEX-6P-1 using *Eco*RI and *Xho*I: 5’-AATTCAAGAAACGTAACCGTCGTCTTTCTGTTGCTC-3’ and 5’-TCGAGAGCAACAGAAAGACGACGGTTACGTTTCTTG-3’. GST-PLK1 expression construct in insect system was a kindly gift from Ray Erickson (Harvard University). To obtain GFP-tagged PLK1, PCR-amplified PLK1 was cloning into pEGFP-C1 by *Eco*RI and *Xho*I digestion. mCherry-LGN plasmid was a gift of Iain Cheeseman. To obtain the His6-NDR1 plasmid for Baculovirus Expression System, PCR-amplified NDR1 cDNA was cloned to pFastaBac^TM^HT B vector (Invitrogen) by recombination method using Mut Express MultiS Fast Mutagenesis Kit (Vazyme).

NDR1 siRNA, whose targeted sequence was the same as described previously^12^, was synthesized by Qiagen. To generate the siRNA-resistant plasmid of NDR1, three bases of targeted sequence were mutated by Quickchange site-directed mutagenesis kit (Agilent Technologies): sr-NDR1 (AAGGCGTGGATTGGGAGCA). All NDR1 mutants (NDR1^K118A^, NDR1^EAIS^, NDR1^3A^ and NDR1^3E^) were constructed by Quickchange site-directed mutagenesis kit based on the siRNA-resistant NDR1 plasmid. All the plasmids were confirmed by sequencing analysis.

### Antibodies

Anti-NDR1 monoclonal antibody (M04) was purchased from Abnova. Anti-pT444-NDR1 rabbit polyclonal antibody was produced and purified as described previously^40^. Anti-Ezrin monoclonal antibody (4A5) was described previously^41^. Other antibodies were used as following: mouse anti-GFP (BD Biosciences); mouse anti-Flag (M2, Sigma); anti-α-tubulin (DM1A, Sigma); rabbit anti-Flag (Sigma, F-7425); rabbit anti-γ-tubulin (Abcam); mouse anti-actin (1A4, Sigma, A-2547); mouse anti-EB1 (BD Biosciences, 610534); mouse anti-Cyclin B (BD Biosciences, 554177); rabbit anti-RFP (Invitrogen, R10367); rabbit anti-Mob1 (Cell Signaling, 3863); rabbit anti-Mob2 (HCCA2) (Abcam, ab103109); rabbit anti-MBP (New England BioLabs, E8032); rabbit anti-pT210-PLK1 (Epitomics, 3646-1); rabbit anti-NuMA (Abcam, ab36999) and ACA (a gift from Dr. Don Cleveland, University of California at San Diego, La Jolla, CA).

### Cell culture, transfection and drug treatments

HeLa, 293 T and LAP-NDR1 stably expressing HeLa (BaCe Bank) cells were maintained in Dulbecco’s modified Eagle’s medium (Gibico) with 10% FBS (Hyclone), 100 IU/ml penicillin and 100 μg/ml streptomycin. For cell synchronization, aliquots of HeLa cells were treated with 2 mM thymidine for 16 hours which allows cells to stay in G1/S. An alternative protocol is to treat cells with 100 ng/ml nocodazole for 18 hours to synchronize cells in prometaphase. For cell treatment, MG132 was used at 20 μM and BI2536 was used at 10 nM. For *in vitro* kinase assay, BI2536 was used at 5 nM. HeLa cells were transfected with constructs or siRNA using Lipofectamine-2000 (Invitrogen), and 293 T cells were transfected with plasmids by calcium phosphates method.

### *In vitro* kinase assay

Assay for NDR1 activity measure: LAP- or Flag-tagged NDR1 wild-type or mutant kinase was purified by GFP-Trap agarose beads (ChromoTek) or Anti-FLAG M2 antibody-agarose beads (Sigma), respectively. Before using, the beads were rinsed by 1X kinase buffer (25 mM Tris-HCl, pH 7.5, 10 mM MgCl_2_, 2 mM dithiothreitol, 50 mM NaCl, 1 mM NaF) containing 0.1% Triton X-100 for three times. GST-tagged NDR1 substrate peptide (GST-SP) was expressed in *E. coli*, purified by glutathione resin and eluted by glutathione. The kinase reaction was performed in 30 μl 1X kinase buffer containing 5 μl bead-bound NDR1 kinase, 3 μg eluted GST-SP, 0.1 μCi [γ-^32^P] ATP, and 50 μM ATP. The mixture was incubated for 30 min at 30 ^o^C followed by centrifugation. The supernatant and precipitant beads were boiled in SDS loading buffer, separated by SDS-PAGE, separately. Then CBB stain or immunoblot was performed.

NDR1 phosphorylated by PLK1: MBP and MBP-tagged NDR1 mutant (K118A or K118A and 3 A) were expressed in *E. coli*, purified by amylose beads and eluted by 10 mM maltose. The kinase reaction was performed in 30 μl 1X kinase buffer containing 0.1 μg PLK1 kinase (Invitrogen), 4 μg eluted MBP protein or MBP-NDR1 mutant (K118A or K118A and 3 A), 0.1 μCi [γ-^32^P]ATP, and 50 μM ATP, at 30 ^o^C for 30 min. The reaction was stopped by 5X SDS loading buffer and separated by SDS-PAGE. Then the gel was stained with CBB.

### Kinase kinetic assay

His-NDR1 fusion protein-expressing Sf9 insect cells were harvested and suspended in His Lysis Buffer (50 mM NaH_2_PO_4_, 300 mM NaCl, 10 mM Immidazole, pH 8.0) containing 0.5% Triton X-100 and 1% protease inhibitor cocktail (Sigma). Then cells were solubilized by sonication on ice and clarified by centrifugation at 13,000 rpm for 20 min. The proteins were purified by incubation with NTA-Ni^2+^ agarose beads for 3 hr. Then the beads were washed with His Lysis Buffer containing 0.5% Triton X-100 and 0.5% protease inhibitor cocktail for three times, His Wash Buffer I (50 mM NaH_2_PO_4_, 300 mM NaCl, 25 mM Immidazole, pH 8.0) for once and His Wash Buffer II (50 mM NaH_2_PO_4_, 300 mM NaCl, 50 mM Immidazole, pH 8.0) for once again. After washes, the protein was eluted by His Elution Buffer (50 mM NaH_2_PO_4_, 300 mM NaCl, 250 mM Immidazole, pH 8.0) and transferred into His Lysis Buffer using PD-10 Desalting Columns (GE Healthcare) for kinase assay.

After the analysis by SDS-PAGE followed by CBB stain, purified soluble His-NDR1 kinases were quantified using ImageJ software. Then His-NDR1 kinases were subjected to an *in vitro* kinase assay using the 10-mer substrate peptide (KKRNRRLSVA) and ATP as the substrates, the ADP assay buffer provided by Amplite^TM^ Universal Fluorimetric Kinase Assay Kit *Red Fluorescence* (AAT Bioquest) as the kinase buffer. The produced ADP was quantified using Amplite^TM^ Universal Fluorimetric Kinase Assay Kit according to the manufacturer’s manuals. The velocity of phosphorylation at a certain concentration of ATP and substrate peptide was calculated from the concentration of produced ADP, the concentration of kinase and the reaction time. Then the kinetic parameters were extracted from various substrate concentrations along with the corresponding velocities of three independent experiments using Michaelis-Menten equation.

### Immunofluorescence, microscopy and data analysis

HeLa cells were plated on sterile, acid-treated glass coverslips and fixed with 3.7% formaldehyde in PBS at 37 ^o^C for 10 min (for staining of NuMA and α-tubulin etc) or ice-cold methanol at -20 ^o^C for 10 min (for γ-tubulin stain). For antibody staining, treated and fixed cells were permeabilized with 0.1% Triton X-100 in PBS for 5 min. Then cells were washed with PBS and blocked by 1% BSA/PBS for 1 hour at room temperature. Then the cells were incubated with primary antibodies for 1 hour at room temperature and with Rhodamine- or Alexa647-conjugated secondary antibodies for 1 hour at room temperature. DNA was stained with DAPI. Coverslips were washed three times with 0.05% Tween-20 in PBS after each antibody incubation or DAPI staining. Coverslips were mounted with Vectasheild Mounting Media (Vector Laboratories).

Images were taken on a DeltaVision microscope (Applied Precision) as previously described^42^. For z-stacks, images were taken at an interval of 0.4 μm by Softworx software (Applied Precision). The single-plane images were deconvoluted using Softworx software (Applied Precision). Then the projection of z-stack images was performed according to the maximum intensity.

For spindle angles calculation, the z-stacks, of which the intensities of γ-tubulin signal reached maximum, were chosen as the sections where spindle poles located. The difference in height (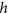) between the two spindle poles was represented as the product of z-stack interval (0.4 μm) and the section number of difference. The distance between the two spindle poles of z-projection (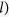 was directly measured by Softworx software.

For the calculation of relative astral microtubule signal intensity, the formula has been described previously^19^. Similar formula was used to calculate the relative cortical fluorescense of LGN and NuMA, and the cortical localization of LGN and NuMA were judged by the threshold value of the relative cortical fluorescense (15% for LGN, 10% for NuMA).The line profile in Fig. 2b was made by Softworx software.

### Time-lapse imaging and quantification

At the beginning of imaging, HeLa cells were maintained at 37 ^o^C in CO_2_-independent media (Gibico) containing 10% FBS (Hyclone) and 1% (2 mM) glutamine (invitrogen). Images were obtained with DeltaVision RT system (Applied Precision). For live mitosis tracking, images of each cell were taken every 4 minutes. For z-stacks, 25 images for each cell were obtained at an interval of 0.8 μm. Images were treated with Photoshop software (Adobe) or ImageJ (NIH) for publication.

The method for spindle angle calculation in time-lapse imaging is similar to that in immunofluorescence. The z-stacks where spindle poles located were judged by the intensities of mCherry-tubulin signals.

### Protein purification and pull-down assay

GST-PLK1 fusion protein was expressed and purified from Sf9 insect cells as described previously^42^. GST and GST-Mob1A were expressed in *E. coli*, purified by glutathione-agarose beads.

For pull-down assay, GST-fusion proteins-bound agarose beads were incubated with purified dissolved MBP-tagged proteins in PBS containing 0.1% Triton X-100 and 0.5 mM DTT at 4^o^C for 3-8 hours. After three washes with PBS containing 0.1% Triton X-100, beads were boiled in SDS sample buffer and separated on 10% SDS-PAGE. The gel was stained with CBB.

## Additional Information

**How to cite this article**: Yan, M. *et al*. Regulation of NDR1 activity by PLK1 ensures proper spindle orientation in mitosis. *Sci. Rep*. **5**, 10449; doi: 10.1038/srep10449 (2015).

## Supplementary Material

Supplementary Information
