# Supplementary material for: Regulation of NDR1 activity by PLK1 ensures proper spindle orientation in mitosis
Source: Sci Rep. 2015 Jun 9;5:10449. doi: 10.1038/srep10449 (PMC4460818; doi:10.1038/srep10449)
Supplement: Supplementary Information [file srep10449-s1.pdf]

Supplementary Information

Regulation of NDR1 activity by PLK1 ensures proper spindle orientation in mitosis

Maomao Yan, Lingluo Chu, Bo Qin, Zhikai Wang, Xing Liu, Changjiang Jin, Guanglan Zhang, Marta Gomez, Alexander Hergovich, Zhengjun Chen, Ping He, Xinjiao Gao, Xuebiao Yao

Supplementary Figure S1

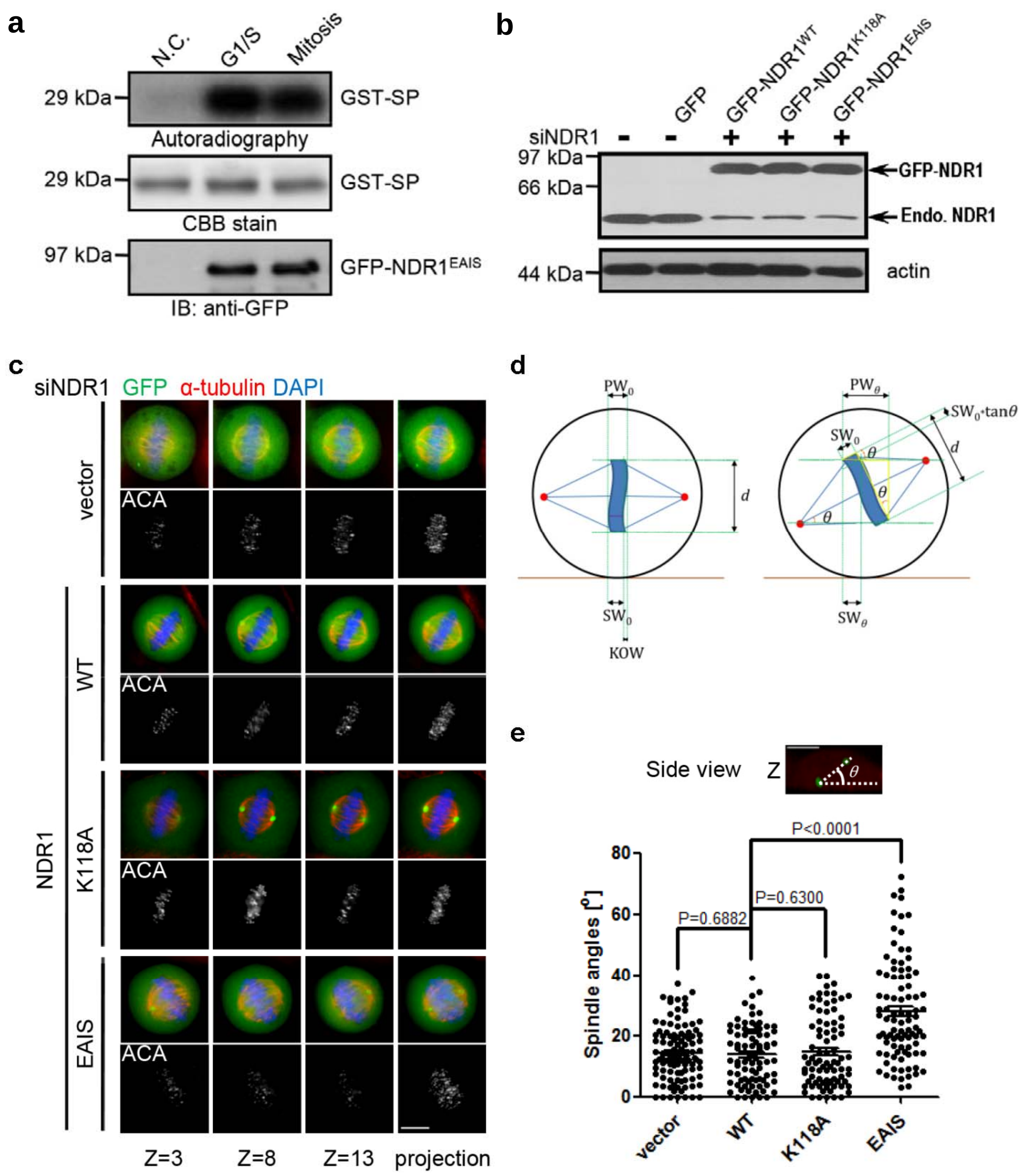

Supplemental Figure S2

Prediction of kinase-specific phosphorylation sites on NDR1

Predicted Sites

ID	Position	Code	Kinase	Peptide	Score	Cutoff
gi|56749457|...	7	T	Other/PLK/P...	*MAMTGSTPCSSMSN	1.596	1.298
gi|56749457|...	183	T	Other/PLK/P...	TLLMKKDTLTEEETQ	1.404	1.298
gi|56749457|...	407	T	Other/PLK/P...	EIKSIDDTSNFDEFP	1.532	1.298

Enter sequence(s) in FASTA format

```
>gi|56749457|sp|Q15208.1|STK38_HUMAN RecName: Full=Serine/threonine-protein kinase 38; AltName:
Full=NDR1 protein kinase; AltName: Full=Nuclear Dbf2-related kinase 1
MAMTGSTPCSSMSNHTKERVTMTKVTLENFYSNLIAQHEEREMRQKKLEKVMEEEGLKDEEKRLRRSAHARKET
EFLRLKRTRLGLEDFESLKVIGRGAFGEVRLVQKKDTGHVYAMKILRKADMLEKEQVGHIRAERDILVEADSLWVV
KMFYSFQDKLNLYLIMEFLPGGDMMTLLMKKDTLTEEETQFYIAETVLAIDSIHQLGFIHRDIKPDNLLLDSKGHVKL
SDFGLCTGLKKAHRTEFYRNLNHSLPSDFTFQNMNSKRKAETWKRNRRQLAFSTVGTPDYIAPEVFMQTGYNKL
CDWWSLGVIMYEMLIGYPPFCSETPQETYKKVMNWKETLTFPPEVPISEKAKDLILRFCCEWEHRIGAPGVEEIKS
NSFFEGVDWEHIRERPAAISIEIKSIDDTSNFDEFPESDILKPTVATSNHPETDYKNKDWVFINYTYKRFEGLTARGA
IPSYMKAAK
```

Supplemental Figure S4

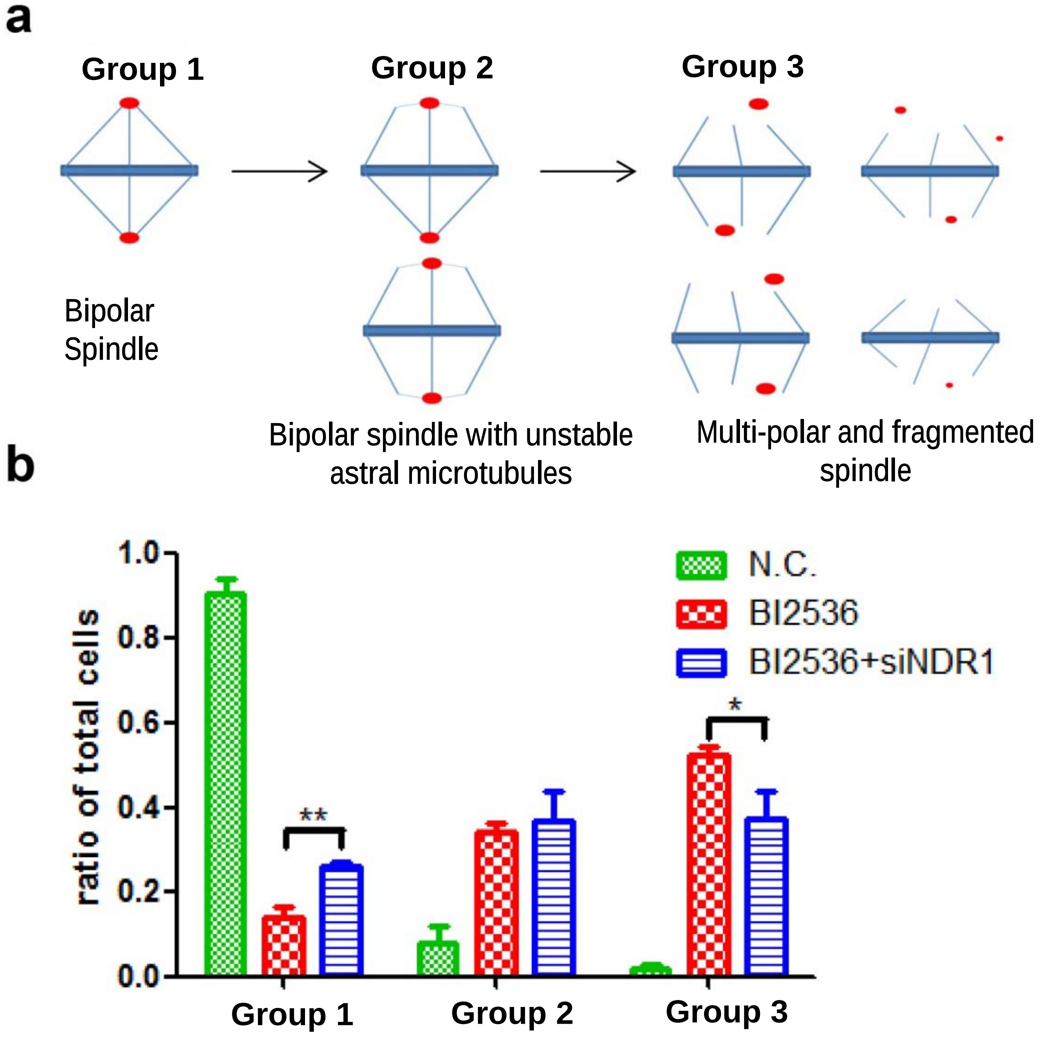

Supplementary Figure S5

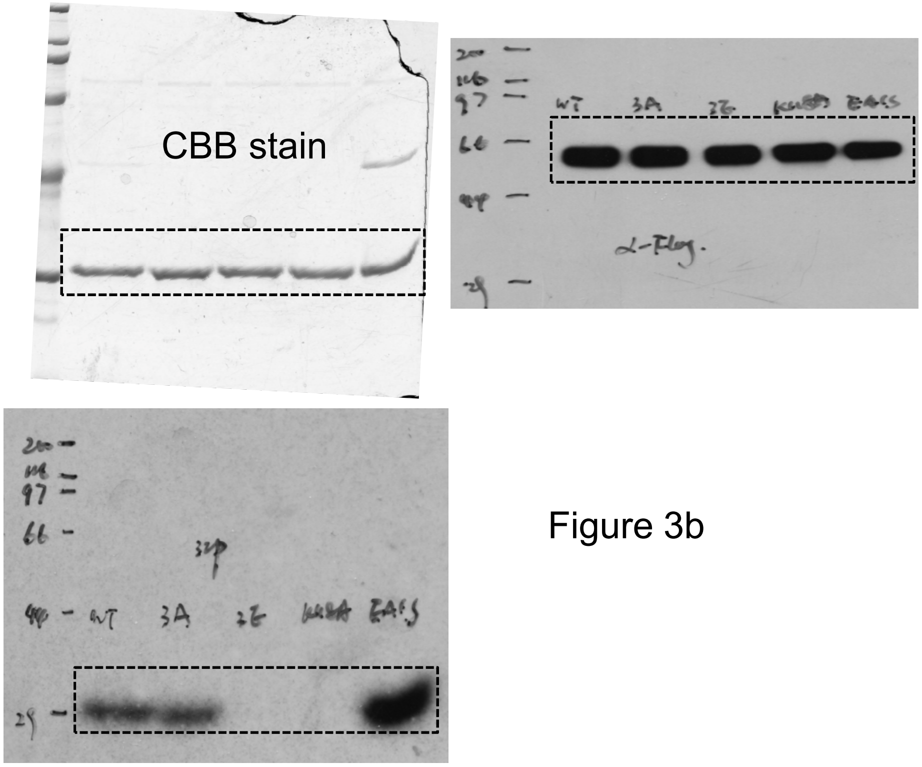

Figure 3b

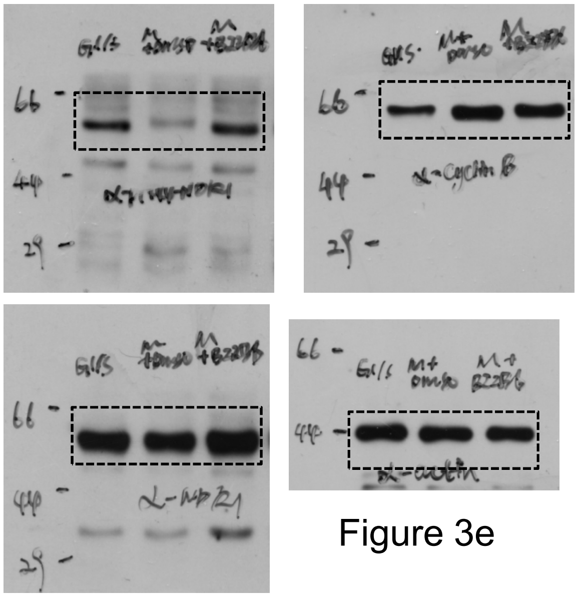

Figure 3e

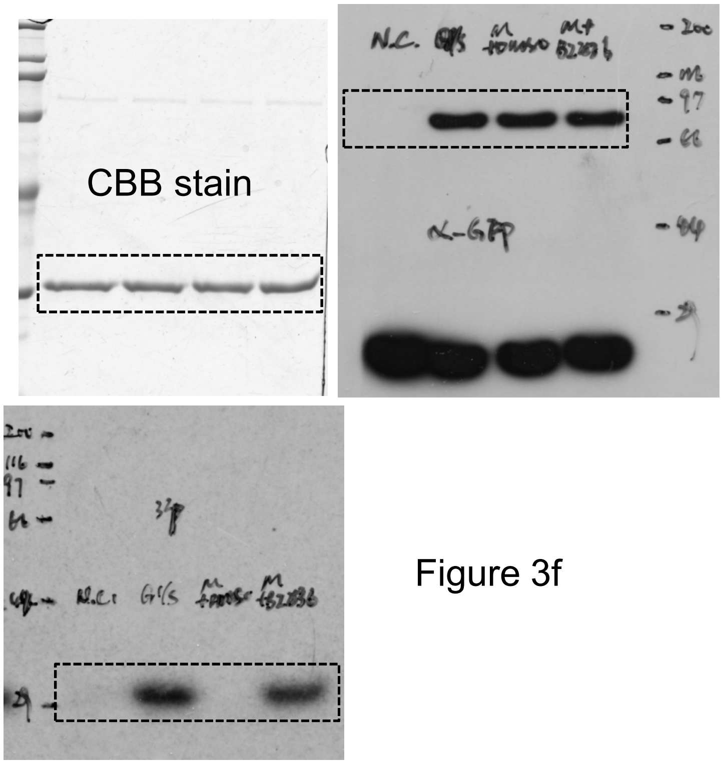

Figure 3f

Supplementary Figure Legends

Supplementary Figure S1. Persistent activation of NDR1 leads to aberrant mitotic spindle orientation

(a) Characterization of the kinase activity of siRNA-resistant GFP-NDR1^{EAIS}. HeLa cells were transfected with GFP vector or siRNA-resistant GFP-NDR1^{EAIS}, and then GFP-NDR1^{EAIS} -transfected cells were synchronized at interphase (G1/S) or mitosis. GFP-NDR1^{EAIS} purified from these cell lysates was subjected to *in vitro* kinase assay with GST-SP as the substrate, in the presence of [γ-^{32}P]-ATP. The activities of GFP-NDR1^{EAIS} were judged and measured by ^{32}P incorporation into GST-SP from *in vitro* kinase assay. The substrate (GST-SP) and kinase (GFP-NDR1^{EAIS}) inputs were tested by Coomassie Brilliant Blue stain and immunoblot, respectively. Cropped gel for CBB stain and autoradiography, cropped blot from another gel.

(a) Characterization of NDR1 siRNA and GFP-tagged NDR1 siRNA-resistant constructs. HeLa cells were transfected with scramble siRNA or scramble siRNA plus GFP or GFP-tagged NDR1 siRNA-resistant constructs, then the whole cell lysates were collected and analyzed by immunoblot with antibodies against NDR1 and actin. Cropped blots from one gel.

(c) Chromosome alignment along with NDR1 activity in immunofluorescence analyses. The projection widths (PW) of chromosome alignment in NDR1^{EAIS}-expressing cells were much larger than the single layer widths (SW) of chromosome alignment, while in NDR1^{WT} or NDR1^{K118A}-expressing cells, the PWs were very close to the SWs. HeLa cells

were co-transfected with NDR1 siRNA plus various siRNA-resistant NDR1 constructs. At 12 hours after transfection, cells were synchronized by 2 mM thymidine for 16 hours, and then released for 10 hours. Before fixation, MG132 was added for 2 hours. Cells were co-stained for microtubules (red), kinetochores (white) and DNA (blue). Images with z-sections (0.4 μm per stack) and z-projection are shown. The scale bar represents 10 μm.

(d) Schematic diagram of the longitudinal section of mitotic cells for explanation of (**c**) Left: When the mitotic spindle axis is parallel to the substratum, the Projection Width (PW) is equal to the addition of the Single layer Width (SW) and the Kinetochore Oscillation Width (KOW). Because the KOW is relatively small compared to SW or PW, the relationships of SW and PW can be expressed as following: $\mathrm{PW}_0 \cong \mathrm{SW}_0$. Right: Because the diameter of chromosome alignmental roundel (*d*) is much bigger than the SW, when the mitotic spindle axis is acclivitous, the PW is much bigger than SW, following:

$\mathrm{PW}_\theta \cong \mathrm{SW}_0/\cos\theta + (d - \mathrm{SW}_0 \times \tan\theta) \times \sin\theta \cong \mathrm{SW}_\theta + (d - \mathrm{SW}_0 \times \tan\theta) \times \sin\theta$. The brown lines represent the substratum, the ring represents the cell cortex, the red dots represent the spindle poles, the blue bands represent the zones of chromosome alignment, the blue lines represent the spindle microtubules, *d* represents the diameter of chromosome alignmental roundel, *θ* represents the spindle angle relative to the substratum, the yellow triangles are right triangles.

(e) The spindle angles of metaphase cells shown in (**c**) were calculated by the formula

described in Figure 1**d** and shown as scatter plots. The positions of spindle poles were roughly judged by the α-tubulin stain. Each point represents one metaphase cell. The mean values are also shown. Bar indicate means ± SEM from analyses of more than 80 cells from three independent experiments. Each *p*-value is calculated using student's *t*-test.

Supplementary Figure S2 Prediction of phosphorylation sites within NDR1 modified by PLK1

Human NDR1 protein sequence in FASTA format was downloaded from UniProtKB and submitted to GPS 2.1 software. The phosphorylated sites on NDR1 were predicted toward PLK1 at the high threshold.

Supplementary Figure S3 Characterization of His-NDR1 purification and the influence of NDR1 activity on PLK1 activity in mitosis

(a) His_6-tagged NDR1 (WT, K118A or 3E) expressed in Sf9 insect cells was purified by NTA-Ni^{2+} agarose beads, after washes, the beads were boiled as "loaded", then kinases were eluted by imidazole. The final solutions were boiled as "eluted". All the samples were separated by SDS-PAGE followed by Coomassie Brilliant Blue stain.

(b) HeLa cells were transfected with scramble siRNA or NDR1 siRNA plus GFP vector or GFP-tagged NDR1 siRNA-resistant constructs. And then cells were synchronized at

mitosis by nocodazole. Mitotic cells were shaked off and boiled for the following immunoblot analysis with the antibodies against NDR1, pT210-PLK1 and tubulin. Cropped NDR1 blot from one gel, cropped other blots from another gel.

Supplementary Figure S4 Illustration of PLK1 chemical inhibitor experimental design and analyses

Supplementary Figure S5 Full-length blots/gels of Figure 3b, e and f
